# The Effect of Dietary Glycemic Properties on Markers of Inflammation, Insulin Resistance, and Body Composition in Postmenopausal American Women: An Ancillary Study from a Multicenter Protein Supplementation Trial

**DOI:** 10.3390/nu9050484

**Published:** 2017-05-11

**Authors:** Violeta Stojkovic, Christine A. Simpson, Rebecca R. Sullivan, Anna Maria Cusano, Jane E. Kerstetter, Anne M. Kenny, Karl L. Insogna, Jessica D. Bihuniak

**Affiliations:** 1 Clinical Chemistry, University of Liège, place du 20-Août, Liège 7 B-4000, Belgium; violeta.s810@gmail.com; 2 Department of Internal Medicine, Section of Endocrinology, Yale University, 300 Cedar Street, New Haven, CT 06510, USA; christine.simpson@yale.edu (C.A.S.); becky.sullivan@yale.edu (R.R.S.); annamaria.cusano@yale.edu (A.M.C.); karl.insogna@yale.edu (K.L.I.); 3 Department of Allied Health Sciences, University of Connecticut, 358 Mansfield Road, Unit 1101, Storrs, CT 06269-1101, USA; jane.kerstetter@uconn.edu; 4 Center on Aging, University of Connecticut Health Center, 263 Farmington Avenue, Farmington, CT 06030, USA; kenny@uchc.edu; 5 Department of Nutrition and Food Studies, Steinhardt School of Culture, Education, and Human Development, 411 Lafayette Street, 5th Floor, New York University, New York, NY 10003, USA

**Keywords:** glycemic index, glycemic load, insulin resistance, body composition

## Abstract

Controversy exists as to whether high glycemic index/glycemic load (GI/GL) diets increase the risk of chronic inflammation, which has been postulated as a pathogenic intermediary between such diets and age-related alterations in body composition and insulin resistance. We conducted an ancillary study to a randomized, double-blind trial comparing the effects of a whey protein supplement (PRO, *n* = 38) and a maltodextrin supplement (CHO, *n* = 46) on bone density to evaluate the impact of a calibrated increase in GI/GL on inflammation, insulin resistance, and body composition in a healthy aging population. Markers of inflammation, HOMA, body composition, and GI/GL (estimated from 3-day food records) were assessed at baseline and 18 months. By 18 months, the GL in the CHO group increased by 34%, 88.4 ± 5.2 → 118.5 ± 4.9 and did not change in the PRO group, 86.5 ± 4.1 → 82.0 ± 3.6 (*p* < 0.0001). Despite this change there were no differences in serum CRP, IL-6, or HOMA at 18 months between the two groups, nor were there significant associations between GL and inflammatory markers. However, trunk lean mass (*p* = 0.0375) and total lean mass (*p* = 0.038) were higher in the PRO group compared to the CHO group at 18 months There were also significant associations for GL and change in total fat mass (*r* = 0.3, *p* = 0.01), change in BMI (*r* = 0.3, *p* = 0.005), and change in the lean-to-fat mass ratio (*r* = −0.3, *p* = 0.002). Our data suggest that as dietary GL increases within the moderate range, there is no detectable change in markers of inflammation or insulin resistance, despite which there is a negative effect on body composition.

## 1. Introduction

A current widely held view is that chronic low-grade inflammation is a major contributor to common chronic diseases, such as cardiovascular diseases [1], diabetes [2], and cancer [3]. Inflammation is also thought to increase with aging [4], which may in part be responsible for the decline in lean mass and increase in adipose tissue that naturally occurs in old age [5]. In this context, low-grade inflammation refers to a variety of mechanisms, including oxidative stress that results in the release of inflammatory cytokines and leads to insulin resistance [6]. Factors that contribute to chronic low-grade inflammation are incompletely understood, but recent attention has focused on the Western diet. In particular, there has been considerable interest in the contribution of glycemic index (GI) and glycemic load (GL) to chronic low-grade inflammation.

The GI is the measure of the glycemic response obtained after the ingestion of a carbohydrate-containing food, compared with a standard (usually glucose or white bread) [7]. The GL is the product of the amount of available carbohydrate in a specific serving size and the GI value (using glucose as the reference food), divided by 100 [8]. It takes into account both quality and quantity of available carbohydrate [9].

Cross-sectional studies in women have reported a positive association between GI/GL and C-reactive protein (CRP) [10,11], a widely used marker of inflammation. However, findings from intervention trials, which have primarily been conducted in overweight and obese individuals, are mixed. Two studies reported a reduction in CRP with lower GL [12] and lower GI diets [13], and two studies found interleukin-6 (IL-6) levels, another commonly used marker of inflammation, to be lower with low GI [14] and low GL [15] diets. Conversely, a number of intervention studies have found no effect of dietary glycemic properties on CRP [16,17,18] or IL-6 [17,18,19].

Data from studies examining the relationship between GI/GL and insulin resistance are also mixed. Results from the Framingham Offspring Cohort suggests a positive association between GI/GL and homeostatic model assessment of insulin resistance (HOMA-IR) [20], while Lau and colleagues reported no relationship [21].

We recently completed a double-blind, randomized, controlled trial (RCT) comparing the effects of a whey protein supplement (PRO) and an isocaloric maltodextrin supplement (CHO) on bone density and body composition in older adults, the SPOON Study (Supplemental Protein to Outsmart Osteoporosis Now; [22]). The study population was relatively homogeneous in usual physical activity, smoking habits, and alcohol consumption and was closely monitored by registered dietitians to ensure that their weight was maintained with the addition of either supplement [22]. We used data from SPOON to undertake an ancillary study to determine the effect of the addition of a fixed glycemic load supplement (PRO or CHO) to the diets of healthy older women on markers of inflammation, insulin resistance, and body composition.

## 2. Materials and Methods

### 2.1. Subjects

Eighty-four post-menopausal women were selected for this ancillary study from SPOON [22]. As noted earlier, the objective of this long-term RCT was to evaluate the impact of a whey protein supplement (Provon^®^ 290, Glanbia Nutritionals, Twin Falls, ID, USA) or isocaloric maltodextrin control supplement (Maltrin^®^ M100, Grain Processing Corporation, Muscatine, IA, USA) on bone mineral density and body composition in postmenopausal women and older men. Subjects were recruited from two study centers (The University of Connecticut Health Center, Farmington, CT, USA, and Yale University School of Medicine, New Haven, CT, USA) between May 2007 and November 2010. Subjects who consumed a minimum of 20 g of either the protein (*n* = 38, protein group, PRO) or isocaloric maltodextrin (*n* = 46, carbohydrate group, CHO) supplement for 18 months were included in this ancillary study.

### 2.2. Dietary Monitoring and Assessment

Methods used to quantify the subjects’ dietary intake in SPOON have been previously described [22]. In brief, at study entry, all participants met with a registered dietitian and were instructed in estimating portion sizes using food models and measuring cups. Prior to each study visit, participants were asked to complete a 3-day food record. Registered dietitians counseled participants to ensure that their weight remained stable during the study. Food records were analyzed using the ESHA Food Processor software program (ESHA Research, Salem, OR, USA, version 10.1.0).

### 2.3. Calculation of Dietary Glycemic Index and Load

The GL of individual food items was assigned according to a publically available database maintained by the University of Sydney (Sydney, Australia; [23]) and published values [8] using glucose as a reference. Serving sizes that had been documented in the subjects’ food records were used to calculate the GL value for the quantity of each food item ingested by the subject. Daily GL was obtained by summing the GL values that accounted for the serving size of each food item consumed over the course of 24 h. Total available carbohydrates were used to calculate daily GI and was calculated by the subtraction of fiber from the total amount of carbohydrate. Daily GI was calculated using a previously published formula, Daily GI = daily GL/total available carbohydrate X 100 [7]. Mean daily GI and GL were calculated for 3 days for each participant at baseline and 18 months.

### 2.4. Sample Analysis

Fasting blood samples were collected by routine venipuncture at baseline and 18 months of SPOON, processed, and stored at −70 °C. Fasting fingerstick glucose measurements were made during SPOON using the Novastrip glucose meter (Yale New Haven Hospital Research Unit, New Haven, CT, USA) or the Bayer Contour glucometer (University of Connecticut Health Center, Farmington, CT, USA). For this ancillary study, all other metabolites were analyzed using previously frozen samples. IL-6 and insulin were measured by the Yale Bone Center Mineral Metabolism Laboratory using R&D Systems High Sensitivity Quantikine ELISA kit (Minneapolis, MN, USA) and Millipore Human Insulin Specific Radio-immunoassay kit (Millipore, St. Charles, MO, USA), respectively. The lower limit of detection for the IL-6 assay was 0.156 pg/mL and for the insulin assay, 2.715 µU/mL. In addition to fingerstick glucose determinations available from SPOON, serum glucose was also measured from stored samples as was high sensitivity-CRP. Both these latter measurements were done in the Yale University Center for Clinical Investigation (YCCI) Core Laboratory using an Alfa Wasserman autoanalyzer (Alfa Wassermann Diagnostic Technologies, West Caldwell, NJ, USA).

### 2.5. Calculation of Insulin Resistance

The HOMA2 calculator, published by the University of Oxford, was used to calculate insulin resistance from fasting insulin and serum glucose. As noted above, serum glucose was analyzed from frozen samples. Glucose is known to be stable in thawed serum samples [24], nonetheless we used fingerstick glucose measurements from our long-term RCT and compared them against serum glucose levels analyzed from frozen samples to validate the stability of the metabolite. A Bland-Altman analysis of 23 simultaneously obtained fingerstick and serum glucose measurements (the latter determined on frozen stored samples) showed a bias of −1.652. We therefore felt comfortable using serum glucose measured on frozen samples in calculating insulin resistance.

### 2.6. Measurement of Body Composition

Body composition in SPOON was measured by DXA, using either a Hologic 4500 W machine (Yale University School of Medicine) or a Lunar Prodigy DPX-IQ (University of Connecticut Health Center) at baseline and 18 months. DXA measurements took place in the morning after an overnight fast.

### 2.7. Ethics

The study was conducted in accordance with the ethical standards of the Investigational Review Boards (IRB#0610001951) at Yale University School of Medicine and University of Connecticut Health Center. All SPOON participants gave their written informed consent.

### 2.8. Statistical Analysis

Data are reported as means ± SEM. The D’Agostino-Pearson omnibus test was used to test data for normality. A Wilcoxon signed-rank test (for non-normally distributed data) or paired *t*-test (for normally distributed data) was used to assess within group differences at baseline vs. 18 months. Between group comparisons were performed using a Mann–Whitney test (for nonparametric data) or unpaired *t*-test (for parametric data). Spearman correlation (for non-normally distributed data) or Pearson correlation (for normally distributed data) analysis was used to examine the relation with dietary GL and change in markers of inflammation, insulin resistance, BMI, and body composition. Data were analyzed using GraphPad Prism 6 (GraphPad Software, Inc., La Jolla, CA, USA). A probability level of *p* < 0.05 was considered statistically significant.

## 3. Results

### 3.1. Study Population

Baseline demographics, anthropometric measures, and dietary and supplement intakes of the study population are summarized in Table 1. Age, weight, body mass index (BMI), and body composition were similar between the two groups at study entry. Study participants were on average within the healthy weight range for their age and height. There were also no differences in baseline energy intake, macronutrient composition of the diet, or estimates of dietary glycemic parameters.

### 3.2. The Effect of the Study Intervention on Dietary Glycemic Load and Glycemic Index and Nutrient Intake

**Figure 1:**
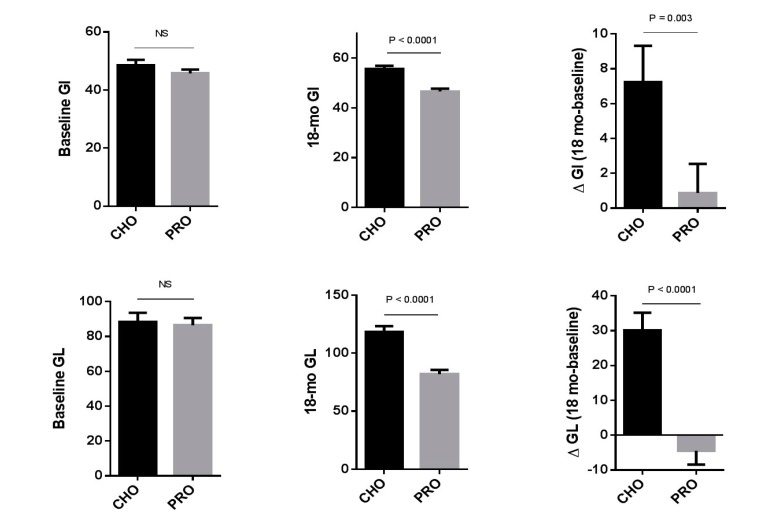
Comparison of 18 month changes in dietary glycemic index and dietary glycemic load in the two study groups (CHO, *n* = 46; PRO, *n* = 38). Between group differences were assessed by unpaired *t*-test (parametric data) or by Mann–Whitney test (nonparametric data). CHO: carbohydrate (maltodextrin) supplement group; GI: glycemic index; GL: glycemic load; NS: non-significant; PRO: protein (whey protein) supplement group.

Over the 18 month study period, the average daily intakes of the maltodextrin and whey protein supplements were 29.5 ± 0.9 g and 30.4 ± 0.9 g, respectively (Table 1). Consumption of the maltodextrin supplement significantly increased both the estimated dietary GI (48.5 ± 2.0 → 55.7 ± 1.2; *p* = 0.0002) and GL (88.4 ± 5.2 → 118.5 ± 4.9; *p* < 0.0001). In contrast, GI (45.7 ± 1.4 → 46.6 ± 1.1) and GL (86.5 ± 4.1 → 82.0 ± 3.6) did not change with the addition of the whey protein supplement. The maltodextrin-induced change in dietary GI and GL resulted in significant differences in dietary glycemic parameters between the two study groups (Figure 1). Despite this, because of regular monitoring by registered dietitians over the course of SPOON, there were no substantive changes in energy or macronutrient intake from food sources (Table 1).

### 3.3. Changes in Markers of Inflammation and Estimates of Insulin Resistance

Although maltodextrin supplementation increased the mean dietary GL of the CHO group by 34%, while protein supplementation resulted in a 5% decline in GL in the PRO group, there were no within or between group differences in markers of inflammation or measures of insulin resistance at 18 months (Table 2). There were also no significant associations between GL and change in markers of inflammation (IL-6: *r* = 0.03, *p* = 0.8; CRP: *r* = −0.03, *p* = 0.8) or insulin resistance (% IR^2^: −0.02, *p* = 0.8).

### 3.4. Changes in BMI and Body Composition

Although the addition of a fixed GL supplement to the usual diets of postmenopausal women for 18 months did not result in changes in markers of inflammation or insulin resistance (Table 2), there were changes observed in body composition. Over the course of the study, lean body mass declined by 0.6 ± 0.3 kg in the CHO group, while it increased by 0.2 ± 0.2 kg in the PRO group (Table 1; *p* = 0.033). These changes resulted in a significant difference in the absolute value for lean body mass at the end of the study (Table 1; *p* = 0.038). Similar and significant changes were also observed for truncal lean mass over the 18-month study period (CHO group: 2.6% decline vs. PRO group: 0.5% increase; Table 1; *p* = 0.007). Furthermore, fat mass increased by 4.7% in the CHO group, although this change did not reach statistical significance (*p* = 0.451). Alterations in body fat were not observed in the PRO group (0%; Table 1; *p* = 0.996). At the end of the study, there was a trend towards a significant difference in change in fat mass between the CHO and PRO groups (Table 1; *p* = 0.08). The change in the lean-to-fat mass ratio was significantly different between the two study groups at 18 months (Table 1; *p* = 0.045), with unfavorable alterations in body composition being observed in the CHO group. There were significant associations for GL and change in total fat mass (*r* = 0.3, *p* = 0.01), change in BMI (*r* = 0.3, *p* = 0.005), and change in the lean-to-fat mass ratio (*r* = −0.3, *p* = 0.002).

## 4. Discussion

This study evaluated the long-term effects of the addition of a fixed GL supplement to the usual diets of postmenopausal women on markers of inflammation, insulin resistance, and body composition. On average, the BMI of our study population was within the healthy range for their age [25]. Maltodextrin supplementation of at least 20 g per day for 18 months resulted in a significant increase in the estimated GI/GL of the diet, but had no effect on markers of inflammation or measures of insulin resistance. However, dietary GL was associated with an unfavorable body composition characterized by higher total fat mass and lower lean-to-fat mass ratio. Lastly, we found an increase in lean mass with a low GL supplement. Our results are consistent with studies that previously reported no association between dietary GI/GL and markers of inflammation and insulin resistance [17,18]. Vrolix and Mensink compared the impact of four different foods with either a high or low GI/GL value on markers of inflammation in 15 overweight men and women for 11 weeks [17]. Consistent with our findings, this earlier study by Vrolix and Mensink [17] observed no changes in CRP or IL-6 when dietary GI/GL were manipulated. Insulin resistance was also unaffected by the various test foods. In both the study by Vrolix and Mensink [17] and the present report, dietitians were employed to ensure that the study interventions did not result in changes in body weight or significant changes in usual dietary intake. Despite the fact that the study by Vrolix and Mensink included a younger, heavier population with metabolic risk factors, CRP values for their study population were similar to ours. Further, the 32 unit difference in GL between the two interventions used by Vrolix and Mensink [17] is nearly identical to the magnitude of change in GL imposed by the addition of the maltodextrin supplement in our current report. Thus, this incremental change in GL does not appear to influence commonly utilized markers of inflammation or insulin resistance in adults of varying weight status. Shikany et al. reported similar results in a younger population of overweight or obese men [18]. That study used a tightly controlled, crossover design with meals provided by a metabolic kitchen and two dietary interventions that differed to a greater extent in their GL value from both the study by Vrolix and Mensink and the current study. There was an approximate 87 unit difference in GL between the two diets utilized by Shikany et al. [18], resulting in a 1.6-fold increase in GL. In contrast, the addition of the maltodextrin supplement in our study led to a 1.3-fold increase in GL.

The relationship between dietary GI/GL and markers of inflammation has also been examined in older adults experiencing, or at higher risk for, chronic diseases thought to be due in part to chronic low-grade inflammation. Bullo et al. conducted a cross-sectional study in 511 older men and women with type 2 diabetes or multiple CVD risk factors that were recruited for the PREDIMED trial [19] and found that levels of tumor necrosis factor (TNF) and IL-6 were higher in the highest quartile of GI at baseline. When subjects were stratified by quartile of GL, there was no association with TNF or IL-6. As noted, we also did not observe an association between GL and markers of inflammation.

Five studies have reported positive associations between dietary GI/GL and markers of inflammation, particularly for IL-6 and CRP, in young and older adults with and without chronic diseases [10,11,26,27,28]. Four of these studies estimated dietary glycemic parameters from food-frequency questionnaires (FFQs) [10,11,19,26], whereas 3-day diet records completed by study subjects after careful instruction by registered dietitians were used to calculate GI/GL for the current study. FFQs tend not to be as precise as food records [29,30], so the strength of the conclusions from these other studies may be limited.

One potential explanation for these varying results is that baseline levels of inflammation vary depending on the study population and, if high, could potentially mask the effect of a change in dietary GI/GL. However, our baseline levels of IL-6 and CRP were similar to the baseline values observed by Bullo [19] and by others [10,11,27] by whom an association of GI/Gl and markers of inflammation were observed.

Although markers of inflammation did not change in our study, we observed favorable changes in body composition with lower GI/GL intakes. Previous studies have reported similar findings [31,32]. Hare-Bruun and colleagues examined the relationship between dietary GI/GL, weight and body composition in 376 middle-aged men and women over a six year period [31]. Higher GI intakes at baseline were associated with increases in body weight, body fat mass, and weight circumference in women at Year 6. In a weight-loss RCT, Maki et al. reported a greater decline in weight and fat mass in overweight and obese men and women receiving a reduced-GL, higher-protein diet compared to a low-fat, portion-controlled diet after a 12-week period of weight loss [32]. Similarly, we observed a similar relationship between dietary GL and fat mass. In the study by Maki et al. [31], lean mass declined in both groups with, surprisingly, the reduced GL/higher protein group experiencing a greater loss of lean mass. In contrast to our study where weight was maintained, lean mass was preserved in subjects receiving a protein supplement and consuming a lower GL diet and declined in those consuming a higher GL supplement and lower protein diet. In the study by Maki et al. [31], subjects in the reduced-GL group had an average protein intake of approximately 1.0 g protein/kg/day, whereas our study subjects in the protein group consumed approximately 1.4 g protein/kg/day. The difference in protein intake may have contributed to the discrepancy in changes in lean mass between the two studies.

The current study had a number of strengths. Supplement adherence and diet were carefully monitored by dietitians during the primary RCT [22]. Details regarding usual physical activity of the study population have been previously described [22]; in brief, subjects did not deviate from their usual level of physical activity for the duration of the study period. Thus, we do not believe physical activity would have confounded our results. Dietary intake and GI/GL were assessed by registered dietitians. Body composition was measured by trained technicians using a widely acceptable and routinely used clinical instrument. We also used multiple biomarkers to assess changes in inflammation. Our study also had limitations. Our study population consisted of normal weight, older adults who were free of most chronic medical conditions and had HOMA-IR values below the insulin resistance threshold of ≥2% [33]. The modest change in dietary glycemic properties did not result in a high dietary GL in the carbohydrate group, and total carbohydrate intake remained moderate (54% of total calories from carbohydrates), and thus may not have been sufficient to induce a change in insulin resistance and inflammation in our healthy older adult population. Our sample size was small. Three-day food records, which were used to estimate dietary intake, are limited in their ability to capture usual intake for all nutrients [34]. Dietary GI and GL were calculated using self-reported dietary data. Because of the nature of the SPOON study design protein intake between the two study groups differed and may in part explain the changes we observed in body composition.

## 5. Conclusions

In summary, we found that the addition of a fixed glycemic load supplement to the diets of otherwise reasonably healthy individuals did not impact markers of inflammation or insulin resistance. However, higher dietary glycemic parameters in healthy older women appear to have unfavorable effects on body composition, and thus, lower GI/GL diets that moderately exceed the RDA for dietary protein should be further explored as a means to help preserve muscle mass and reduce fat mass with aging.

## Figures and Tables

**Table 1:** Demographics, anthropometric indices, and dietary intake at baseline and 18 months ^1^.

	CHO			PRO		
	Baseline	18 Months	Δ (18 Months-Baseline)	Baseline	18-Months	Δ (18 Months-Baseline)
*N*	46			38		
Age	69.3 ± 0.9			68.9 ± 0.9		
Weight (kg)	66.5 ± 1.5	66.8 ± 1.6	0.3 ± 0.6	68.1 ± 1.7	68.0 ± 1.6	−0.2 ± 0.4
BMI (kg/m^2^)	25.8 ± 0.6	26.0 ± 0.6	0.2 ± 0.2	26.0 ± 0.6	26.0 ± 0.6	0.009 ± 0.1
Body lean mass (kg)	39.0 ± 0.6	38.4 ± 0.6 ^a^	−0.6 ± 0.3 ^a^	40.1 ± 0.7	40.3 ± 0.7 ^a^	0.2 ± 0.2 ^a^
Trunk lean mass (kg)	19.6 ± −0.3	19.1 ± −0.3 ^a,b^	−0.5 ±0.2 ^a^	20.0 ± 0.3	20.1 ± 0.4 ^a^	0.1 ± 0.1 ^a^
Body fat mass (kg)	25.5 ± 1.1	26.7 ± 1.2	1.2 ± 0.5	25.9 ± 1.3	25.9 ± 1.2	−0.009 ± 0.4
Lean/Fat	1.7 ± 0.1	1.6 ± 0.1	−0.1 ± 0.03 ^a^	1.7 ± 0.1	1.7 ± 0.1	−0.001 ± 0.03 ^a^
Dietary Calories (kcal)	1661 ± 51.6	1626 ± 55.5	−34.5 ± 54.9	1627 ± 45.6	1551 ± 57.1	−70.4 ± 53.5
Total Calories (kcal)	1661 ± 51.6	1729 ± 55.5	68.2 ± 54.5	1627 ± 45.6	1678 ± 58.0	56.5 ± 54.2
Dietary Carbohydrate (g)	201.2 ± 6.9	202.8 ± 8.7	1.6 ± 7.5	207.2 ± 9.0	198.9 ± 8.9	−8.6 ± 8.4
Fiber (g)	19.4 ± 1.0	18.9 ± 1.0	−1.1 ± 1.1	21.0 ± 1.2	18.3 ± 1.0	−2.8 ± 1.4
Supplement Carbohydrate (g)	0	29.5 ± 0.9		0		
Total carbohydrate (g)	201.2 ± 6.9	232.3 ± 8.7 ^a,b^	31.1 ± 7.4 ^a^	207.2 ± 9.0	198.9 ± 8.9 ^a^	−8.6 ± 8.4 ^a^
Dietary Protein (g)	71.5 ± 2.2	69.8 ± 2.5	−1.7 ± 2.3	73.5 ± 2.7	68.3 ± 2.5	−5.1 ± 2.9
Supplement Protein (g)	0			0	30.4 ± 0.9	
Total protein (g)	71.5 ± 2.2	69.8 ± 2.5 ^a^	−1.7 ± 2.3 ^a^	73.5 ± 2.7	98.5 ± 2.8 ^a,b^	25.1 ± 3.1 ^a^
Dietary Fat (g)	62.5 ± 3.9	57.1 ± 2.8	−5.4 ± 3.7	56.1 ± 2.7	51.6 ± 2.5	−4.4 ± 3.2

^1^ Values are averages ± SEMs. Values with a superscript letter: ^a^ significantly different between the two study groups, *p* < 0.05 by Mann–Whitney test (nonparametric data) or unpaired *t*-test (parametric data); ^b^ significantly different from baseline, *p* < 0.05 by Wilcoxon signed-rank test (nonparametric data) or paired *t*-test (parametric data). BMI: body mass index; GI: glycemic index; GL: glycemic load; *N*: sample size.

**Table 2:** Markers of inflammation and insulin resistance ^1^.

		CHO			PRO		
		Baseline	18-Months	Δ	Baseline	18-Months	Δ
IL-6 (pg/mL)		1.7 ± 0.2	1.9 ± 0.2	0.2 ± 0.2	1.5 ± 0.1	1.8 ± 0.1	0.3 ± 0.1
CRP (mg/L)		1.5 ± 0.2	2.0 ± 0.2	0.5 ± 0.2	1.9 ± 0.2	2.5 ± 0.4	0.6 ± 0.3
HOMA	% B ^2^	132.8 ± 7.1	117.1 ± 6.2	−15.7 ± 8.4	137.0 ± 8.6	133.3 ± 6.1	−3.7 ± 9.3
	% S ^2^	75.0 ± 4.5	75.7 ± 4.5	0.66 ± 4.7	66.9 ± 3.6	64.1 ± 3.3	−2.9 ± 2.6
	% IR ^2^	1.6 ± 0.1	1.5 ± 0.1	−0.02 ± 0.08	1.7 ± 0.1	1.7 ± 0.1	0.02 ± 0.07

^1^ Values are averages ± SEMs. % B: β-cell function; CRP: C-reactive protein; HOMA: Homeostasis Model Assessment; IL-6: Interleukin 6; % IR: Insulin resistance; % S: Insulin sensitivity. ^2^ Values estimated as percentages of a normal reference population.
